# Stewardship Challenges, PK/PD Evidence, and Prescribing Appropriateness for Recently Approved Antibiotics in Pediatric Practice

**DOI:** 10.3390/antibiotics15090883

**Published:** 2026-09-09

**Authors:** Alessandra Romandini, Chiara Resnati, Stefania Crucitta, Stefano Agliardi, Federico D’Amico, Giulia Angela Carla Pattarino, Elena Altieri, Romano Danesi, Costantino De Giacomo

**Affiliations:** 1 Clinical Pharmacology Unit, Chemical-Clinical Analyses Laboratory, A.S.S.T Grande Ospedale Metropolitano Niguarda, 20162 Milan, Italy; chiara.resnati@ospedaleniguarda.it (C.R.);; 2 Division of Pediatrics, A.S.S.T Grande Ospedale Metropolitano Niguarda, 20162 Milan, Italycostantino.degiacomo@ospedaleniguarda.it (C.D.G.); 3 Oncology & Onco-Hematology Department, Università degli Studi di Milano, 20162 Milan, Italy

**Keywords:** antibiotics, pediatric, pharmacology, multidrug-resistant, antimicrobial stewardship, long-acting lipoglycopeptides

## Abstract

The rise of multidrug-resistant (MDR) pathogens in the pediatric population represents a significant global health challenge, compounded by a historically stagnant antibiotic pipeline for children. While several novel antibiotics have been approved for adults in recent decades, pediatric labeling is often deferred due to the complexities of developmental pharmacology and due to a certain precautionary prudence in introducing new drugs onto the market for this population. This review explores the landscape of the most recent antibiotics, including advanced cephalosporins (ceftaroline, cefiderocol, and ceftobiprole), novel beta-lactam/beta-lactamase inhibitor combinations (e.g., ceftazidime/avibactam, meropenem/vaborbactam), and long-acting lipoglycopeptides. We analyze their approval trials, pediatric-specific PK/PD profiles, and the balance between on-label use and evidence-based off-label prescriptions. Furthermore, we emphasize the role of antimicrobial stewardship through the “3 D’s” rule and the evolution of Therapeutic Drug Monitoring (TDM) from reactive safety controls to proactive, model-informed precision dosing (MIPD). Finally, we advocate for a multidisciplinary synergy between pediatricians and pharmacologists as the cornerstone for optimizing outcomes and preserving the efficacy of the future antibiotic pipeline.

## 1. Introduction

### 1.1. A Dire Need

Rising antimicrobial resistance represents one of the most consequential threats to child health worldwide. Over the past two decades, the incidence and clinical impact of multidrug-resistant (MDR) bacterial infections in pediatric populations have increased across different geographic regions and care settings [1]. Carbapenem-resistant Enterobacterales (CRE) [2], extended-spectrum beta-lactamase–producing (ESBL) Enterobacterales [3], and methicillin-resistant *Staphylococcus aureus* (MRSA) [4] now account for a growing share of severe community-acquired and healthcare-associated infections in neonates, infants, and older children [5].

These organisms are associated with higher morbidity and mortality, longer hospital stays, greater need for intensive care and invasive support, and substantially increased healthcare costs, compared with susceptible pathogens. In low- and middle-income countries the burden is worsened by limited access to diagnostics, intensive care, and newer therapeutic agents, producing stark disparities in outcomes between regions [6]. Despite the rising need for new therapeutic agents in the pharmacological armamentarium to treat MDR infections in younger patients, a persistent “pediatric gap” exists in the antimicrobial approval pipeline.

### 1.2. The Pediatric Gap

Historically, antibiotics, just like most classes of drugs, are developed and approved first in adult populations, with pediatric indication and/or formulations often following years later.

Nonetheless, over the past 30 years, regulatory requirements have required and incentivized pediatric research: the FDA enacted the Pediatric Research Equity Act in 2003, and the EMA established the Paediatric Regulation in 2007. Regulatory agencies have been using a scheme colloquially known as the “carrot and the stick” approach: they require pediatric studies for certain new drugs and biologic products, as well as new indications that are likely to be used in children, while sponsors and pharmaceutical companies also may apply for incentives, such as a 6-month extension of market exclusivity for the drugs for voluntarily conducting requested pediatric trials (in the United States), as well as a 2-year extension of market exclusivity for pediatric orphan drugs that fully comply with the EMA’s Paediatric Investigation Plan (in Europe) [7,8].

Nevertheless, many new agents still lack a timely pediatric pharmacokinetic (PK) profile and pediatric safety data and dosing data at the time of adult approval, and they are often granted a deferral for pediatric studies until there are sufficient data to demonstrate the safety and efficacy of the products in an adult population; this happens, in practice, in over 80% of cases [9]. This time lag means clinicians often must rely on extrapolated adult data, off-label use, or compassionate-use programs to treat children with MDR infections. The consequence is either delayed access to potentially superior agents or empiric use of older, less safe drugs with suboptimal efficacy.

And while extrapolation of adult efficacy data remains important in minimizing the need for pediatric efficacy trials, indication-specific, dose-finding and safety pediatric trials are still crucial for offering the best standard of care in the youngest patients, who deserve population-specific, high-quality evidence.

Pediatric trials aside, many challenges to precise pediatric dosing definition remain. From a developmental standpoint, the maturation of absorption, distribution, metabolism, and excretion (ADME) systems varies markedly from the preterm neonate through adolescence, necessitating age-stratified PK and pharmacodynamic (PD) studies.

Finally, the practical challenges—such as limited blood volume available for PK sampling and lower prevalence of target infections in single centers—complicate trial logistics and increase the need for novel trial designs, such as sparse sampling, population PK modeling, and adaptive trials, as well as international multicenter collaboration [10].

The convergence of a rising pediatric MDR burden, delayed pediatric access to new antimicrobials, and unique developmental pharmacology creates an urgent need for strategies that accelerate safe pediatric evaluation and access. These strategies include earlier inclusion of pediatric cohorts, at minimum during late efficacy or phase IIIb trials (to also monitor potential pediatric-specific safety concerns); use of innovative PK/PD algorithms and modeling approaches to inform dosing; and harmonization of regulatory requirements across jurisdictions and countries.

Nevertheless, it should be acknowledged that antibiotics, especially the most recent ones, are expensive and often not available in many countries; these settings would benefit from a global-scale action to favor the distribution at lower cost of salvage therapies in selected severe cases.

The present expert opinion focuses on antibiotics that have been recently approved for use in the pediatric population, with the aim of providing a clinically relevant framework to support their appropriate positioning and inform therapeutic decision-making. This work reviews the available evidence for recently approved antibiotics, discusses their potential roles across relevant pediatric infectious diseases, and highlights key considerations in selecting the most appropriate agent in different clinical scenarios.

## 2. Advanced Cephalosporins

### 2.1. Ceftaroline Fosamil

Ceftaroline is known as a novel “fifth-generation” cephalosporin that shows in vitro bactericidal activity against MRSA [11], and is also active against other common bacteria in the pediatric population, such as *S. pneumoniae*, *S. pyogenes*, *Haemophilus influenzae* and *Moraxella catarrhalis* [12]. Nevertheless, it does not exhibit activity against Gram-negative bacteria producing ESBL or carbapenemase [13].

It was initially approved in the European Union and the United States for the treatment of adults with complicated skin and soft tissue infection (cSSTI)/acute bacterial skin and skin structure infection (ABSSSI) and community-acquired pneumonia (CABP) [14,15]. These approvals were subsequently extended, based on additional pediatric studies conducted as part of the PIP, to include pediatric patients aged ≥2 months and, more recently, neonates (for ABSSSI, only in the United States) [14,15]. European labeling has recently been further extended to include high-dose recommendations for pediatric patients aged ≥2 months with cSSTI [15].

Ceftaroline has undergone extensive clinical trials in pediatric patients (birth to ≤18 years), including single-dose PK studies and multiple-dose safety and efficacy studies, though the trials’ primary endpoint was safety, and they were not powered for comparative interferential efficacy analysis. Across phase II/III-IV trials in patients aged ≥2 months to <18 years with CABP, complicated CABP, or ABSSSI, ceftaroline fosamil demonstrated clinical efficacy generally similar to standard comparator treatments, as well as similar adverse event incidence and therapeutic failure rates [16]. No new safety concerns were identified, and patterns of treatment-emergent adverse events were similar to those reported in a pooled analysis of the trials in adults [17]. In a phase II, open-label, non-comparative trial in children <2 months of age with late-onset sepsis, the safety and tolerability of ceftaroline fosamil were consistent with previous data in older children and adults [18].

A recent study carried out on a population of 20 children with cystic fibrosis showed, through 160 measured serum concentrations and 119 measured urine concentrations, that pediatric patients with cystic fibrosis have a higher level of ceftaroline clearance than those without, and may therefore benefit from higher doses, though no standard regimen has been established yet [19]. Further studies would be useful in establishing a specific optimal dosing, as *S. aureus* is the most commonly isolated organism in the early course of the disease and therefore ceftaroline may be an interesting candidate for such populations.

In line with other beta-lactam antibiotics, the percentage of time that free-drug concentrations are above the bacteria MIC during a dose interval (%fT > MIC) has been shown to be the PK/PD index associated with efficacy [20].

In Europe, adult and pediatric high-dose regimens with longer 2 h infusions are approved for patients aged ≥2 months with cSSTI caused by rare *S. aureus* isolates with ceftaroline fosamil MICs of 2–4 mg/L [15]. In addition, the European label, but not the United States label, includes recommended ceftaroline dose adjustments for pediatric patients with impaired kidney function (estimated creatinine clearance ≤ 50 mL/min) [15]. Finally, a high-dose regimen (15 mg/kg, or 600 mg if weighing > 40 kg, infused over 120 min every 8 h) for treatment of hematogenously acquired *S. aureus* osteomyelitis has been studied in children aged 1 to 17 years [21], though this indication is currently off-label.

In fact, ceftaroline has long been used, both in adult and pediatric populations, for off-label indications, such as bacteremia, osteomyelitis, septic arthritis, sepsis, endocarditis, meningitis, device infections, and nosocomial pneumonia [22] with clinical success, though data sufficient to constitute a strong supportive framework is still lacking.

Nevertheless, ceftaroline represents a paradigm shift within the beta-lactam class, representing a unique agent approved for use in patients from birth, with extensive pediatric data and with a broad spectrum of activity ranging all the way to MRSA, though still maintaining the safety profile of a cefalosporine.

### 2.2. Ceftobiprole

Ceftobiprole medocaril is a broad-spectrum fifth-generation cephalosporin with activity against both Gram-positive and Gram-negative bacteria. Its activity against MRSA is mediated by high-affinity binding to PBP2a, and its spectrum also includes *Pseudomonas aeruginosa*, *Streptococcus pneumoniae*, and *Enterococcus faecalis*. This dual coverage supports its use in selected severe infections in which simultaneous activity against resistant Gram-positive and Gram-negative pathogens is clinically relevant [23].

As with other beta-lactams, the primary pharmacodynamic determinant of ceftobiprole’s efficacy is the percentage of the dosing interval during which free-drug concentrations remain above the minimum inhibitory concentration (%fT > MIC). The conservative PK/PD target used in pediatric development was based on maintaining free-drug concentrations above an MIC of 4 mg/L, corresponding to the EUCAST non-species-specific PK/PD breakpoint [24,25].

Pediatric phase III and pharmacokinetic (PK) studies have included neonates, infants, children, and adolescents. Across these studies, ceftobiprole showed low levels of protein binding, minimal metabolism, and predominantly renal elimination. As in adults, peak plasma concentrations were achieved at the end of infusion, and accumulation after repeated administration was minimal because of its relatively short elimination half-life [24,26].

A randomized phase III trial involving hospitalized patients aged 3 months to <18 years with CAP or HAP found ceftobiprole has a clinical response comparable to standard-of-care cephalosporins [27]. This led to support for the pediatric use of ceftobiprole. It has been demonstrated that pediatric exposure profiles are generally comparable to those observed in adults. Across pediatric age groups, median half-life values ranged from approximately 1.9 to 2.9 h, whereas overall exposure overlapped substantially with adult values. Importantly, the lowest observed %fT > MIC remained above 50%, suggesting adequate PK/PD target attainment throughout childhood [26].

Population pharmacokinetic modelling subsequently integrated data from patients from birth to 17 years of age. Body weight and renal function were identified as the main determinants of inter-individual variability. Model-informed simulations suggest that 15 mg/kg every 12 h in neonates and infants younger than 3 months, and every 8 h in older pediatric patients, leads to exposures comparable to those observed in adults, while maintaining adequate PK/PD target attainment [25,28].

Despite encouraging clinical and PK data, several limitations remain. Pediatric development has primarily focused on pneumonia, leaving limited to no evidence for invasive infections such as bacteremia, endocarditis, osteoarticular infections, and infections occurring in critically ill children receiving extracorporeal support. Furthermore, many pediatric dosing recommendations rely on exposure matching and pharmacometric extrapolation from adult populations, rather than large efficacy-driven trials [25].

Overall, ceftobiprole is among the advanced cephalosporins most extensively characterized for pediatric use. Its combined anti-MRSA and antipseudomonal activity, favorable safety profile, and robust PK/PD characterization make it a valuable option when both spectrum components are clinically justified [29,30,31]. Nevertheless, its clinical positioning is less defined in the most complex clinical settings. When available, TDM should be performed to tailor antibiotic therapy to each specific case and in order to gather further clinical information [25,26].

Ceftobiprole use in pediatric settings remains limited by its approval (only >3 months of age), the lack of pediatric data in most complex clinical settings and the report of increased mortality if used for VAP, though it holds great value for its spectrum of activity, which extends from MRSA to *P. aeruginosa*.

Table 1 further clarifies the comparative characteristics to guide antimicrobial choice in pediatric clinical practice for ceftaroline and ceftobiprole, which have overlapping indications.

### 2.3. Cefiderocol

Cefiderocol is a novel siderophore-conjugated cephalosporin that utilizes a unique “Trojan horse” mechanism, exploiting bacterial active iron transport systems to penetrate the outer membranes of multidrug-resistant (MDR) and carbapenem-resistant Gram-negative pathogens. As a time-dependent antibiotic, its primary PD driver is the percentage of the time the free-drug concentration remains above the minimum inhibitory concentration (%fT > MIC) [32]. Data from the PEDI-CEFI phase 2 trial (NCT04335539) indicate that weight-adjusted dosing—60 mg/kg (<34 kg) or 2000 mg (≥34 kg), via a 3 h infusion every 8 h—achieves steady-state plasma exposures in patients aged 3 months to <18 years comparable to those in adults. These regimens consistently maintain trough concentrations above susceptibility breakpoints, ensuring a high probability of target attainment (100% fT > MIC) [33,34].

Further investigations, including the APEKS-PEDI [35] and the neonatal NEO-CEFI [36] studies, are ongoing to refine dosing across pediatric subpopulations, with model-based approaches highlighting the importance of postmenstrual age for PK optimization in neonates [35,36].

Regarding safety, cefiderocol appears to be well tolerated in children, with a profile consistent with the beta-lactam class. Systematic reviews and trial data report no drug-related serious adverse events, significant hepato-renal toxicity, or clinical laboratory abnormalities [33,37]. One unique, benign pediatric phenomenon is the “red wine urine syndrome”, resulting from cefiderocol’s interaction with iron from blood products [38]. Collective evidence from case reports and systematic reviews suggests that cefiderocol is an effective off-label salvage therapy for life-threatening carbapenem-resistant infections [39,40,41,42], even during extracorporeal support (ECMO/CRRT) [43], though clinicians should remain vigilant regarding pre-existing resistance among metallo-β-lactamase producers and the risk of microbiological relapse in complex settings [44,45], as well as the fact that currently its use remains off-label, and the clinical trials and data collection are still ongoing. In fact, given its wide spectrum, cefiderocol should be used only as off-label salvage therapy in suspicion of highly resistant Gram-negative infections.

## 3. Novel Protected Beta-Lactam Combinations

### 3.1. Ceftolozane/Tazobactam and Ceftazidime/Avibactam

Ceftolozane/tazobactam (C/T) and ceftazidime/avibactam (CAZ/AVI) provide activity against *P. aeruginosa*, including MDR and difficult-to-treat resistant (DTR) phenotypes, and are used when conventional antipseudomonal beta-lactams are no longer reliable [46,47]. However, they should not be considered interchangeable: C/T is primarily a potent antipseudomonal agent, with ceftolozane retaining activity against isolates with AmpC overexpression, efflux pump upregulation, and OprD loss, whereas CAZ/AVI provides a broader beta-lactamase inhibition profile, particularly against class A carbapenemases such as KPC and class D OXA-48-like enzymes, although remaining inactive against MBLs unless combined with aztreonam [48,49]. In CAZ/AVI, activity against resistant *P. aeruginosa* is mainly driven by avibactam-mediated inhibition of relevant beta-lactamases, including AmpC-derived cephalosporinases [46,47]. These differences are clinically relevant in children with severe infections, especially in ICU settings, where beta-lactam exposure may be difficult to predict due to factors such as augmented renal clearance, expanded volume of distribution, fluid shifts, or extracorporeal support [50,51].

Both agents have followed the traditional pediatric development pathway for new antimicrobials. For C/T, randomized pediatric trials evaluated C/T versus meropenem in complicated urinary tract infections (cUTI) and C/T plus metronidazole versus meropenem in complicated intra-abdominal infections (cIAI), supporting weight-based regimens in neonates, infants, children and adolescents [52,53]. A dedicated neonatal and young-infant phase I subgroup analysis further suggested that single-dose PK profiles were generally comparable to those of older children and that the drug was well tolerated in this vulnerable population, although the small number of enrolled subjects limits definitive conclusions [54].

For CAZ/AVI, pediatric development includes a phase I PK study, two randomized phase II trials in children aged ≥3 months to <18 years with cUTI and cIAI, and population PK analyses supporting exposure matching with adults [55,56,57,58]. In Europe, CAZ/AVI has also been approved from 3 months of age for cUTI, cIAI, HAP including VAP, bacteremia associated with these infections, and infections due to aerobic Gram-negative organisms with limited treatment options, depending on local regulatory wording [59]. This broader formal indication makes CAZ/AVI particularly relevant when the expected pathogen is a carbapenem-resistant *Enterobacterales* rather than isolated DTR *P. aeruginosa*.

For both C/T and CAZ/AVI, dosing in children is driven by time-dependent PD, with %fT > MIC as the key exposure parameter. Adequate inhibitor exposure is also essential in beta-lactam/beta-lactamase inhibitor combinations to maintain beta-lactam activity. Therefore, dosing cannot rely on body weight alone, especially in critically ill children, who may require higher PK/PD targets and are at risk of reduced target attainment due to augmented renal clearance or extracorporeal support [51,60].

For C/T, age-based and weight-based dosing strategies for cUTI and cIAI are supported by both population PK analyses and pediatric trials [52,53,61]. For CAZ/AVI, population PK modeling suggests that pediatric regimens can achieve exposures comparable to those in adults, but target attainment may be challenging in critically ill patients, especially when renal clearance is high or when the MIC approaches the susceptibility breakpoint [58]. In these circumstances, prolonged or continuous infusion and TDM, when available, may be useful adjuncts in optimizing exposure, although pediatric outcome data remain limited.

Adult comparative evidence remains more extensive than pediatric data for DTR *P. aeruginosa*, with pediatric experience mainly derived from case reports, series, stewardship cohorts, and reviews. C/T has been used as rescue therapy for MDR *P. aeruginosa* infections in children, including bloodstream infections, endocarditis, and cystic fibrosis pulmonary exacerbations, due to its potent antipseudomonal activity [62,63,64]. Reduced susceptibility to C/T and CAZ/AVI has been reported in cystic fibrosis isolates even without previous exposure, supporting isolate-specific susceptibility testing rather than reliance on expected activity [45]. CAZ/AVI has broader pediatric real-world experience, particularly in carbapenem-resistant infections and immunocompromised patients [59,65].

The main limitation of the pediatric evidence is that registrational trials generally enrolled relatively stable children with cUTI or cIAI and were not designed to evaluate outcomes in the patients in whom these agents are most needed: neonates, critically ill children, patients receiving extracorporeal support, children with cystic fibrosis, hemato-oncologic patients, and those with bloodstream, CNS, bone, joint, or endovascular infections. Much of the evidence in these scenarios remains observational and vulnerable to confounding by indication, concomitant therapy, and source control. A 2026 pediatric systematic review and evidence map found that most available studies were case reports or case series, while randomized trials excluded critically ill children and carbapenem-resistant infections; clinical and microbiological cure rates were generally high, but the certainty of evidence remains limited [65].

C/T finds its pediatric clinical positioning in children with infections suspected to be MDR *P. aeruginosa*, or ESBL-producing, while CAZ/AVI focuses on resistant *Enterobacterales* (especially ESBL-producing, or CRE).

Table 2 further clarifies the comparative characteristics to guide antimicrobial choice in pediatric clinical practice for ceftolozane/tazobactam, ceftazidime/avibactam and cefiderocol, which have overlapping indications.

### 3.2. Carbapenem-Based Combinations

#### 3.2.1. Imipenem/Cilastatin/Relebactam

Imipenem/cilastatin/relebactam (IMI/REL) is a combination of a carbapenem, a renal dehydropeptidase-I inhibitor, and a novel diazabicyclooctane β-lactamase inhibitor designed to protect imipenem from degradation by Ambler class A (including KPC) and class C (AmpC) β-lactamases, while also restoring activity against certain imipenem-resistant *P. aeruginosa* strains [69,70]. Pediatric PK and PD evidence has recently advanced through phase Ib and phase II/III trials, which collectively established weight-based dosing regimens from birth to <18 years designed to achieve target exposures, and guided its FDA approval in the United States for pediatric populations subsequent to birth [71,72,73,74].

In these studies, imipenem achieved a geometric mean %fT > MIC of 56.5–93.7%, consistently surpassing the ≥30% target (at an MIC of 2 μg/mL), while relebactam exposures exceeded the PK/PD target of a free-drug AUC/MIC ratio ≥ 8.0 [73]. Geometric mean AUC_0–24_ values for imipenem and relebactam ranged from 610 to 795 μM/h and 399 to 634 μM/h, respectively, across pediatric cohorts [74]. These PK/PD data support the clinical efficacy observed against multidrug-resistant (MDR) and KPC-producing *Enterobacterales*, where IMI/REL maintains high in vitro susceptibility (93.5–100% for KPC-positive isolates) [69,70,71,72,73,74]. Regarding safety, IMI/REL was generally well-tolerated across all pediatric age groups, with a profile comparable to active comparators and consistent with adult safety data [73,74,75]. The majority of reported adverse events were mild to moderate in intensity—most commonly, gastrointestinal symptoms such as vomiting and diarrhea—and no study-related deaths or significant new safety signals were identified [75]. These data confirm that weight-based dosing provides a robust PK/PD profile and a safety margin that supports its therapeutic role in treating pediatric resistant infections [73,74,75].

Imipenem/cilastatin/relebactam finds its pediatric clinical positioning in suspicion of *Enterobacterales*, especially in ESBL- or KPC-producing strains.

#### 3.2.2. Meropenem/Vaborbactam

Meropenem/vaborbactam (MER/VAB) is a combination of a carbapenem and a cyclic boronic acid-based beta-lactamase inhibitor specifically designed to inhibit carbapenemases such as KPC, thereby protecting meropenem from degradation and restoring its activity against CRE [76,77]. PK characterization in the pediatric population (ranging from 3 months to 18 years) indicates that both moieties follow two-compartment models with first-order elimination, where body weight and creatinine clearance serve as significant covariates [77]. To achieve a probability of target attainment (PTA) ≥ 90%, current modeling supports a dosing regimen of 40 mg/kg (up to 2 g) administered as a 3.5 h intravenous infusion every 8 h for children aged ≥3 months; for neonates and infants under 3 months, extrapolated data suggest a dose of 20 mg/kg [77]. Real-world PK data from a 4-year-old patient confirmed a volume of distribution of 0.59 L/kg and a clearance of 13.1 mL/min/kg, achieving 100% target attainment (40%fT > MIC) with a 6 h dosing frequency [78]. Regarding safety, clinical evidence from pediatric case series and reports—including patients treated for bloodstream infections and intra-abdominal abscesses—indicates the drug is well-tolerated, with no identified adverse drug events or complications reported during treatment or follow-up [77,78]. Ongoing phase II/III trials, specifically, TANGO-KIDS, VABOR-KIDS (NCT06828848), and ML-VAB-201-3248-2 (NCT06672978), are currently further evaluating the safety, PK profile, and tolerability of the drug in children with complicated urinary tract infections, with further results expected in the near future [79,80,81].

Meropenem/vaborbactam is particularly interesting in pediatric clinical practice for its activity against CRE, though its use in children is still off-label, as it does not yet have pediatric approval.

### 3.3. Aztreonam/Avibactam

Aztreonam/avibactam (ATM/AVI) is a fixed-dose combination of a monobactam and a non-β-lactam β-lactamase inhibitor specifically engineered to circumvent resistance in metallo-β-lactamase (MBL)-producing Gram-negative bacteria [82]. At present, clinical data regarding the use of ATM/AVI in pediatric patients are extremely limited. A primary prospective, randomized trial designed to evaluate its efficacy and safety in serious MBL-producing infections (NCT03580044) supported the regulatory filing, along with REVISIT, though the study under-enrolled, and, according to its final results, enrolled no subjects under 18 years of age [83]. Despite this clinical gap, microbiological surveillance through the INFORM Program (2019–2023) demonstrates that ATM/AVI is highly potent against pediatric isolates, inhibiting >99.9% of *Enterobacterales* at an MIC ≤ 8 mg/L across various infection types, including pneumonia and UTIs [84]. From a PK/PD perspective, the current dosing framework utilizes a loading dose followed by 3 h extended infusions to maximize the time-above-MIC, with maintenance doses adjusted every 6 to 8 h based on renal function (creatinine clearance) [83,85]. The ongoing study NCT05639647 is currently recruiting pediatric participants (9 months to <18 years) to characterize essential population PK parameters, including AUC, clearance, and volume of distribution [86]. Regarding safety, evidence from the adult cohort in NCT03580044 showed that 91.7% of subjects experienced treatment-emergent adverse events (TEAEs), with 41.7% experiencing serious adverse events (SAEs), although many were associated with the underlying disease severity [83]. For the pediatric population, the NCT05639647 protocol specifically monitors the proportion of participants reporting liver injury and acute kidney injury, alongside clinically significant laboratory and vital sign abnormalities, to establish a robust safety profile across different age cohorts [85]. Ongoing pediatric development is guided by the EMA PIP and regulatory requirements to bridge these data gaps and establish formal dosing for children [86]. 

Aztreonam/avibactam finds its promising pediatric clinical positioning when aerobic Gram-negative bacterial infection and/or production of metallo-beta-lactamases are suspected, as salvage therapy in patients with no other option.

Table 3 further clarifies the comparative characteristics to guide antimicrobial choice in pediatric clinical practice for imipenem/cilastatin/relebactam, meropenem/vaborbactam and aztreonam/avibactam, which have overlapping indications.

## 4. Long-Acting Lipoglycopeptides

Long-acting lipoglycopeptides are increasingly relevant in the treatment of pediatric infectious diseases, as their prolonged half-lives may simplify parenteral treatment, reduce the burden of repeated intravenous administration, and support earlier discharge in selected patients with Gram-positive infections [87,88,89].

Between the two agents, dalbavancin has the more robust evidence base, including pediatric population pharmacokinetic modeling, PK/PD target attainment analyses, a phase 3 ABSSSI study, systematic review data, and real-world case series suggesting early discharge and reduced hospitalization [88,89,90]. Oritavancin is also pharmacologically interesting, and its use is supported by its recent approval in Europe, but its off-label use in children remains under-studied and under-reported due to comparable clinical outcome evidence being still limited [89].

### 4.1. Dalbavancin

Dalbavancin is the long-acting lipoglycopeptide with the most substantial pediatric evidence base for outpatient parenteral use. Its main pharmacologic advantage is its prolonged half-life, which translates into its only needing few and far-apart administrations; this characteristic makes it particularly suited for a reduction in intravenous treatment burden and shortening hospitalization in favor of outpatient treatment [90]. The recommended dose of dalbavancin is a single dose based on the patient’s age and weight: 22.5 mg/kg up to 6 months of age, and 18 mg/kg between 6 months and 18 years of age (maximum 1500 mg).

The pediatric pharmacokinetic study included 43 children aged 3 months to 11 years and analyzed 311 concentration samples using a three-compartment population pharmacokinetic model. Simulations identified pediatric regimens that achieved adult-like exposure, supporting the feasibility of adult-referenced PK/PD target attainment in children [87,88].

This pharmacologic rationale is supported by clinical data. In the pediatric phase 3 ABSSSI study, favorable clinical response at 48–72 h was observed in 97.4% of children receiving the single-dose regimen and 98.6% of those receiving the two-dose regimen, with no new safety concerns [90]. Real-world pediatric reports are consistent with this positioning. A 2024 case series of five children with ABSSSI described dalbavancin as effective and emphasized its role in facilitating early discharge and reducing healthcare burden [91]. Another 2024 report found dalbavancin to be a safe, effective, and a convenient alternative in selected children with complicated non-ABSSSI infections [92]. A more recent pediatric series suggested that dalbavancin may also be useful when inpatient or outpatient parenteral therapy is difficult to maintain [93].

Overall, dalbavancin can be positioned in pediatric practice as the long-acting lipoglycopeptide with the most mature body of integrated PK, PK/PD, and clinical data. Its greatest practical value is evident in ABSSSI/cSSTI and in selected consolidation strategies where simplified dosing may reduce the length of stay, facilitate outpatient parenteral therapy, and limit the need for prolonged vascular access [87,88,90,91].

### 4.2. Oritavancin

Oritavancin shares the same conceptual appeal as dalbavancin because it is also a long-acting lipoglycopeptide with sustained activity against Gram-positive pathogens. However, its activity spectrum extends to vancomycin-intermediate *Staphylococcus aureus* (VISA) and vancomycin-resistant *Staphylococcus aureus* (VRSA). Its terminal half-life is approximately 245 h, and its recommended dose is 15 mg/kg given as a single dose via intravenous infusion over 3 h (maximum 1200 mg), and recently a 1 h infusion [89].

However, the pediatric off-label use evidence remains much thinner than the evidence for dalbavancin’s off-label use. The main pediatric trial that led to its approval in Europe was the ORKIDS pharmacokinetic study, which evaluated a single 15 mg/kg intravenous dose in children and showed that patients aged 6 to <18 years had pharmacokinetic profiles similar to adults, whereas children aged 2 to <6 years had lower exposure than the adult target range [89]. 

Oritavancin should be considered as a valid first option in treating ABSSSI in children, especially in VISA and VRSA.

Table 4 further clarifies the comparative characteristics to guide antimicrobial choice in pediatric clinical practice for dalbavancin and oritavancin, which have overlapping indications.

## 5. Prescriptive Appropriateness and Positioning

### On-Label vs. Off-Label: Navigating the Legal and Clinical Evidence Framework

Despite regulatory efforts, off-label prescribing remains common in pediatric antimicrobial therapy, particularly in cases involving severe infections, rare pathogens, neonates, and newly introduced agents. Its use may be justified when supported by a sound pharmacological rationale and the best available evidence. Treatment decisions should consider microbiological susceptibility, PK/PD data, disease severity, age-appropriate dosing, organ function, and safety monitoring [94,95].

A practical approach to prescribing novel antibiotics in children should combine the traditional principles of antimicrobial stewardship with pediatric pharmacology. Pediatric stewardship programs have been associated with reductions in inappropriate antibiotic use, costs, and antimicrobial resistance, and professional societies emphasize that antibiotics should be used only when necessary and, in such cases, with the appropriate agent, dose, duration, and route.

Pharmacokinetic models, TDM and early involvement of clinical pharmacologists can be additional tools for better adapting the therapeutical choice, especially in extremely complex clinical subsets such as augmented renal clearance, organ dysfunction, extracorporeal membrane oxygenation (ECMO), or continuous renal replacement therapy (CRRT).

## 6. Antimicrobial Stewardship in Pediatrics

### 6.1. The 3 Right D’s: Right Drug, Right Dose, Right Duration

The “3 D’s” rule remains a practical framework for pediatric antimicrobial stewardship, emphasizing selection of the right drug, dose, duration, and route to optimize outcomes while minimizing adverse consequences. Drug selection should consider likely pathogens, local resistance patterns, site penetration, toxicity, formulation, and patient-specific factors [96,97]. Dose optimization is particularly challenging, because developmental physiology and clinical conditions substantially affect PK, supporting individualized dosing strategies [98,99]. Appropriate treatment duration is equally important in reducing unnecessary drug exposure, microbiome disruption, and antimicrobial resistance, with regular reassessment and early discontinuation when appropriate representing key stewardship practices [100].

### 6.2. Diagnostic Stewardship: Integrating Rapid Molecular Diagnostics with Bedside Prescribing

Diagnostic stewardship is a key component of pediatric antimicrobial stewardship, as appropriate prescribing relies on accurate and timely microbiological diagnosis. These complementary strategies improve diagnostic accuracy and optimize antimicrobial use [101]. Stewardship teams play a central role in integrating clinicians, microbiologists, pharmacologists and laboratory specialists to translate diagnostic information into appropriate treatment decisions [102]. 

In practice, diagnostic stewardship involves selecting the right test for the right patient at the right time, avoiding unnecessary testing, and ensuring appropriate interpretation of microbiological results, including rapid molecular diagnostics.

### 6.3. Reactive vs. Proactive TDM

TDM in pediatric infectious diseases has evolved from a toxicity-prevention tool into a precision-medicine strategy aimed at optimizing both efficacy and safety. Modern approaches focus on identifying underexposure and achieving PK/PD targets associated with antimicrobial activity [103,104]. Given the high PK variability in children due to age, inflammation, organ function, critical illness, and supportive therapies, TDM is increasingly integrated with physiologic, microbiological, and concentration data to guide individualized treatment [105].

The distinction between reactive and proactive TDM represents a major shift in pediatric clinical pharmacology. Reactive TDM is performed after concerns such as toxicity, lack of efficacy, or abnormal concentrations arise, whereas proactive TDM is implemented early in patients at high risk of PK target non-attainment due to age, clinical instability, organ dysfunction, or high PK variability [106]. This approach moves beyond toxicity prevention toward MIC-targeted efficacy, assessing whether the drug exposure is sufficient to achieve the desired PK/PD target according to pathogen susceptibility. This is particularly relevant in critically ill children, where underexposure may remain undetected until treatment failure occurs [106].

Model-informed precision dosing (MIPD) is a major innovation in pediatric antimicrobial optimization, combining population PK models, patient-specific characteristics, and measured drug concentrations to provide individualized dosing recommendations. Through Bayesian forecasting, prior PK predictions are updated with real-world patient data to optimize therapy [98,104]. Studies suggest that MIPD improves target attainment compared with conventional dosing, often requiring dose or infusion adjustments, highlighting the limitations of standard regimens in achieving PK/PD targets in real-world pediatric practice [98,107].

The future development of MIPD is likely to depend on even more advanced modeling approaches. Tanaka et al. (2025) argue that PBPK-based approaches may further improve MIPD by incorporating developmental physiology and, potentially, target-site exposure [105,108]. Additional feasibility data from pediatric intensive care units suggest that MIPD is transitioning from a theoretical concept to a practical clinical tool. Its role may be even more relevant in pediatrics than in adults due to the limited amount of pharmacological data and greater PK variability in the latter. The combination of TDM and reliable prediction software is increasingly recognized as a valuable support for routine antibiotic optimization beyond research settings [107].

Microsampling represents a promising technology for pediatric TDM and pharmacokinetic research, addressing the limitations of standard sampling strategies arising due to limited blood volume, repeated venipuncture distress, and logistical challenges [109,110]. Techniques such as dried blood spots and volumetric absorptive microsampling allow quantitative analysis from very small blood volumes, although further validation of blood-to-plasma relationships and therapeutic ranges is needed [109,110,111]. From a stewardship perspective, microsampling is important not only for reducing the procedural burden, but also because it may enable wider implementation of proactive TDM and precision dosing in settings where conventional repeated sampling is impractical. Moreover, it may streamline monitoring pathways and offer potential economic advantages [109,110,111,112].

An important consideration must be acknowledged when mentioning futuristic approaches to antimicrobial stewardship: their cost may hinder their widespread use in secondary and tertiary hospitals, and they are—or will be—available only in a small number of specialized centers worldwide. Centers without the capability to monitor antibiotic concentrations and explore more advanced approaches would benefit from the practical algorithm in Table 5.

### 6.4. The Pediatrician–Pharmacologist Synergy

The increasing complexity of pediatric antimicrobial therapy makes the partnership between pediatricians and pharmacologists increasingly important. Antibiotic choice, dose optimization, TDM interpretation, Bayesian forecasting, and PK/PD target assessment all require the integration of bedside clinical judgment with advanced pharmacologic expertise [104,106].

This synergy becomes even more critical in critically ill children, where rapidly changing organ function, inflammation, extracorporeal therapies, and fluid shifts may alter drug exposure in ways that are difficult to anticipate using conventional dosing alone [106,107].

Table 6 overviews novel antibacterial agents in pediatric clinical practice.

## 7. The Future Pipeline and Conclusions

In March 2026, the World Health Organization (WHO) published three new target product profiles (TPPs) for antibacterial agents targeting severe MDR infections, including Gram-negative and Gram-positive infections and bacterial meningitis, in high-risk populations [113]. The 2025 analysis of antibacterial agents in clinical and preclinical development identified 90 agents in development, but only 15 were considered innovative [114]. These TPPs emphasize the need for new antimicrobials addressing underserved populations, including immunosuppressed patients, the elderly, children, neonates, and patients with complex infections.

Currently, the antibacterial pipeline offers grounds for cautious optimism, but it still falls short of the urgent clinical needs of children, especially neonates and infants with multidrug-resistant infections. 

Among the investigational agents in advanced clinical development, cefepime/taniborbactam is particularly interesting, as it targets metallo-β-lactamase-producing organisms [115], and its pediatric investigational plan (for Europe) and initial pediatric study plan (for the United States) have been approved by the regulatory agencies, are underway and include pediatric patients down to newborns [115]. Zosurabalpin, a first-in-class tethered macrocyclic peptide has been developed to treat carbapenem-resistant *Acinetobacterm baumannii* (CRAB) [116], and its pediatric investigational and study plans have been approved and grantedin a deferral. Among the agents currently being studied, sulbactam/durlobactam also represents an important pipeline candidate, as it targets resistance mechanisms that are especially relevant in neonatal sepsis, including carbapenem resistance [115], while the new, interesting antibiotics classes that may revolutionize future antibiotic research and use are bacteriophages and antibodies [114]. Bacteriophages are viruses that selectively infect and kill bacteria, representing a promising alternative to traditional antibiotics and an ally against antimicrobial resistance; their selective mechanism of action would only target bacteria responsible for an infection, leaving the beneficial bacterial flora intact, unlike wide-spectrum antibiotics. Monoclonal antibodies also would have an extremely selective mechanism of action, reducing collateral damage to the host microbiome relative to traditional wide-spectrum antibiotics. On the other hand, the same high specificity also represents a major limitation, especially when dealing with variable targets such as bacterial polysaccharidic structures. Thus, current strategies focus on creating monoclonal antibodies cocktails, or on generating antibodies against protein antigens conserved across different pathogenic species, or combination-based approaches utilizing antibiotics.

Future antibiotic research should therefore shift from a sequential model, in which pediatric studies begin only after adult development is complete, to a parallel model that incorporates pediatric pharmacokinetics, safety, dosing, and formulation work much earlier. The WHO specifically emphasized that pediatric antibiotic development lags behind adult development by nearly a decade and that lack of investment, weak trial infrastructure, and missing child-friendly formulations continue to slow access. For pediatric populations, especially neonates, this means trials should be designed around age- and weight-specific cohorts, clinically meaningful endpoints, and formulations suitable for real-world use in both high-resource and resource-limited settings [115].

In conclusion, the use of antibiotics in pediatrics is very widespread, even for newly introduced agents for which real-world clinical data are still often incomplete or lacking. Moreover, further new agents are planned for commercialization in the coming years, though often they may not be studied in pediatrics. Additionally, the pediatric patient is often very complex due to variability in clinical conditions, which differ from one child to another and can rapidly change over time, significantly impacting pharmacokinetics. Addressing these matters is essential for ensuring that children may at least benefit from the same modern scientific and therapeutic advances as their adult counterparts, hopefully tailored to their unique needs.

## Figures and Tables

**Table 1:** Ceftaroline vs. ceftobiprole: comparative characteristics to guide antimicrobial choice in pediatric clinical practice [14,15,28]. Abbreviations: CAP = community-acquired pneumonia, CNS = central nervous system, CrCl = creatinine clearance, EMA = European Medicines Agency, ESBL = extended-spectrum beta-lactamase, FDA = Food and Drug Administration, GA = gestational age, HAP = hospital-acquired pneumonia, KPC = Klebsiella pneumoniae carbapenemase, MDR = multidrug-resistant, MRSA = methicilline-resistant *Staphylococcus aureus*, NA = not available/not approved, PNA = postnatal age, RCT = randomized controlled trial, SSTI = skin and soft tissue infection, VAP = ventilator-associated pneumonia, VISA = vancomycin intermediate *Staphylococcus aureus*, VRE = vancomycin-resistant *Enterococci*, VRSA = vancomycin-resistant *Staphylococcus aureus*.

Characteristics	Ceftaroline	Ceftobiprole
FDA/EMA approval date	2010/2012	2024/2013
Drug class	Fifth-generation cephalosporine	Fifth-generation cephalosporine
Type of bactericidal activity	Time-dependent	Time-dependent
Efficacy index	%fT > MIC	%fT > MIC
Known coverage	*S. aureus* (also MRSA, VISA, VRSA), and methicillin-resistant coagulase-negative *Staphylococci*; *S. pneumoniae* (including MDR and penicillin-resistant), beta-hemolytic *Streptococci*, *H. influenzae*, *Moraxella catarrhalis*, *Klebsiella*	*S. aureus* (also MRSA, VISA, VRSA), *S. pneumoniae* (also reduced penicillin sensitivity), coagulase-negative *Staphylococci*, AmpC beta-lactamases-producers, *H. influenzae*, *Moraxella catarrhalis*, *E. faecalis*, *P. aeruginosa*
Main Target Genotypes	MRSA, VRE (limited activity)	MRSA, VRE (limited activity)
Approval age class	Birth (GA ≥ 34 weeks/PNA ≥ 12 days)–18 years	>3 months–18 years
Known resistance gaps	Limited activity vs. *Enterococci*, ESBL producers, AmpC-producers, *P. aeruginosa*, carbapenemase-producers (KPC, *Acinetobacter*).	ESBL-producing *Enterobacterales*, carbapenemase-producers (KPC, *Acinetobacter*)
Approved pediatric indications	SSTIs (including MRSA), CAP (not MRSA, >2 months)	CAP (not MRSA)
Type of evidence	RCT	RCT
Specific warnings	Diarrhea, including *C. difficile* infection; seroconversion from a negative to a positive direct Coombs’ test	Increased mortality if used for VAP; seizures and other CNS effects; diarrhea, including *C. difficile* infection
Infusion time	1 h	2 h
Main elimination route	Renal	Renal
Renal adjustment	Yes (CrCl < 50 mL/min)	Yes (CrCl < 50 mL/min)
Hepatic adjustment	No	No
Drug-drug interactions	No	No
Summary of clinical positioning	MRSA, approved birth–18 yrs, extensive pediatric data	MRSA and *P. aeruginosa*, approved >3 months-18 yrs, lacking data in most complex clinical settings

**Table 2:** Ceftolozane/tazobactam vs. ceftazidime/avibactam vs. cefiderocol: comparative characteristics to guide antimicrobial choice in pediatric clinical practice [66,67,68]. Abbreviations: cIAI = complicated intra-abdominal infection, CNS = central nervous system, CrCl = creatinine clearance, cUTI = complicated urinary tract infection, EMA = European Medicines Agency, ESBL = extended-spectrum beta-lactamase, FDA = Food and Drug Administration, GA = gestational age, HAP = hospital-acquired pneumonia, KPC = *Klebsiella pneumoniae* carbapenemase, MDR = multidrug-resistant, MRSA = methicilline-resistant *Staphylococcus aureus*, NA = not approved, PNA = postnatal age, RCT = randomized controlled trial, VAP = ventilator-associated pneumonia, VRE = vancomycin-resistant *Enterococci*.

Characteristics	Ceftolozane/Tazobactam	Ceftazidime/Avibactam	Cefiderocol
FDA/EMA approval date	2014/2015	2015/2016	2019/2020
Drug class	Fifth-generation cephalosporine/beta-lactamase inhibitor	Third generation-cephalosporine/non-beta-lactam beta-lactamase inhibitor	Siderophore cephalosporine
Type of bactericidal activity	Time-dependent	Time-dependent	Time-dependent
Efficacy index	%fT > MIC	%fT > MIC	%fT > MIC
Known coverage	MDR *P. aeruginosa*, *Enterobacterales*	*Enterobacterales*, (*P. aeruginosa* as alternative agent)	*Enterobacterales*, *P. aeruginosa*, *A. baumannii*, *S. maltophilia*, *B. cepacia*
Main Target Genotypes	ESBL	AmpC, ESBL, KPC, OXA-48	AmpC, ESBL, KPC, OXA-48, MBL, NDM
Approval age class	GA ≥ 32 weeks and PNA ≥ 7 days	>3 months	NA
Known resistance gaps	MRSA, VRE	MRSA, VRE	Most Gram-positive and anaerobic bacteria
Approved pediatric indications	cIAI, cUTI and pyelonephritis	cIAI, cUTI, HAP/VAP (EMA only)	NA (in adults: cUTIs, HAP/VAP)
Type of evidence	RCT	RCT	Case reports
Specific warnings	Kidney toxicity; diarrhea, including *C. difficile* infection	Seizures and other CNS effects; diarrhea, including *C. difficile* infection	Higher all-cause mortality in critically ill patients with MDR Gram-negative HAP/VAP, bacteremia and sepsis; seizures and other CNS effects; hypokalemia
Infusion time	1–2 h	2 h	3 h
Main elimination route	Renal	Renal	Renal
Renal adjustment	Yes (CrCl < 50 mL/min)	Yes (CrCl < 50 mL/min)	Yes (CrCl < 60 mL/min or >120 mL/min)
Hepatic adjustment	No	No	No
Drug-drug interactions	No	Probenecid can decrease elimination of avibactam	No
Summary of clinical positioning	Suspicion of MDR *P. aeruginosa* infection, also ESBL	Resistant *Enterobacterales* (ESBL, CRE)	Salvage therapy in suspicion of highly resistant Gram-negative infection (NA < 18 years)

**Table 3:** Imipenem/cilastatin/relebactam vs. meropenem/vaborbactam vs. aztreonam/avibactam: comparative characteristics to guide antimicrobial choice in pediatric clinical practice [75,79,86]. Abbreviations: cIAI = complicated intra-abdominal infection, CNS = central nervous system, CrCl = creatinine clearance, cUTI = complicated urinary tract infection, EMA = European Medicines Agency, ESBL = extended-spectrum beta-lactamase, FDA = Food and Drug Administration, HAP = hospital-acquired pneumonia, KPC = *Klebsiella pneumoniae* carbapenemase, MBL = metallo-beta-lactamases, MDR = multidrug-resistant, MRSA = methicilline-resistant *Staphylococcus aureus*, MRSE = methicilline-resistant *Staphylococcus epidermidis*, NA = not approved, RCT = randomized controlled trial, VAP = ventilator-associated pneumonia, VRE = vancomycin-resistant *Enterococci*.

Characteristics	Imipenem/Cilastatin/Relebactam	Meropenem/Vaborbactam	Aztreonam/Avibactam
FDA/EMA approval date	2019/2020	2017/2018	2025/2024
Drug class	Carbapenem/dehydropeptidase inhibitor/beta-lactamase inhibitor	Carbapenem/beta-lactamase inhibitor	Monobactamic/non-beta-lacram beta-lactamase inhibitor
Type of bactericidal activity	Time dependent	Time dependent	Time dependent
Efficacy index	%fT > MIC	%fT > MIC	%fT > MIC
Known coverage	*Enterobacterales*, *P. aeruginosa*, *H. influenzae*, *E. faecalis*	*Enterobacterales* (also CRE)	*Enterobacterales*, *S. maltophilia*, (*P. aeruginosa* as alternative agent)
Main Target Genotypes	AmpC, ESBL, KPC	AmpC, ESBL, KPC	AmpC, ESBL, KPC, OXA-48, MBL
Approval age class	Birth to ≤ 18 years	NA	NA
Known resistance gaps	MRSA, MRSE, *Enterococcus faecium*, NDM, VIM, IMP, OXA-48	MBL, OXA-48, carbapenem-resistant *A. baumannii*, *P. aeruginosa*, or *S. maltophila*	Anaerobic bacteria, Gram-positive
Approved pediatric indications	FDA: cUTI and pyelonephritis (in patients with no other option), cIAI (in patients with no other option), HAP/VAP; EMA: Pending	NA (cUTI and pyelonephritis in adults)	NA (cIAI, HAP/VAP, cUTI and pyelonephritis, other aerobic Gram-negative infections, as salvage therapy in adults)
Type of evidence	RCT and concluded phase II/III trials	Ongoing phase II/III trials	Ongoing phase II/III trials
Specific warnings	Seizures and other CNS symptoms (especially when associated with ganciclovir)	Seizures and other CNS symptoms; diarrhea, including *C. difficile* infection; rhabdomyolisis	Serious skin disorders (e.g., toxic epidermal necrolysis; diarrhea, including *C. difficile* infection
Infusion time	30 min	3 h	3 h
Main elimination route	Renal	Mainly renal	Renal
Renal adjustment	Yes (CrCl < 60 mL/min)	Yes (CrCl < 50 mL/min)	Yes (CrCl < 50 mL/min)
Hepatic adjustment	No	No	No
Drug–drug interactions	Valproic acid concentrations may be decreased when associated	Effectiveness of hormonal contraceptives (containing a progestine and an estrogen) may be reduced	Probenecid may increase avibactam concentration
Summary of clinical positioning	When suspicion of Enterobacterales, potentially ESBL or KPC	When suspicion of CRE	When suspicion of aerobic Gram-negative infection and/or MBL, in patients with no other option

**Table 4:** Dalbavancin vs oritavancin: comparative characteristics to guide antimicrobial choice in clinical practice [87,88,89,90,91]. Abbreviations: ABSSSI = acute bacterial skin and skin structure infection, CrCl = creatinine clearance, EMA = European Medicines Agency, FDA = Food and Drug Administration, MRSA = methicilline-resistant *Staphylococcus aureus*, RCT = randomized controlled trial, VISA = vancomycin intermediate *Staphylococcus aureus*, VRE = vancomycin-resistant *Enterococci*, VRSA = vancomycin-resistant *Staphylococcus aureus*.

Characteristics	Dalbavancin	Oritavancin
FDA/EMA approval date	2014/2015	2014/2015
Drug class	Lipoglycopeptide	Lipoglycopeptide
Type of bactericidal activity	Concentration-dependent	Concentration-dependent
Efficacy index	AUC/MIC	AUC/MIC
Known coverage	Gram-positive, including MRSA, *E. faecalis* and *E. faecium*	Gram-positive, including MRSA, *E. faecalis* and *E. faecium*
Main target genotypes	MRSA, VRE	MRSA, VISA, VRSA
Approval age class	Birth to ≤18 years	EMA: >3 months–18 yrs FDA: NA
Known resistance gaps	VISA (higher MICs), VanA	VanA, VanB (VRE underexplored)
Approved pediatric indications	ABSSSI and/or sepsis	ABSSSI
Type of evidence	RCT	RCT
Specific warnings	Diarrhea, including *C. difficile* infection; risk of otovestibular toxicity when associated with other ototoxic drugs	Concomitant use of warfarin; interference with certain laboratory coagulation tests; diarrhea, including *C. difficile* infection; risk of otovestibular toxicity when associated with other ototoxic drugs
Infusion time	30 min	3 h (recentlyapproved a newer formulation for 1 h infusion)
Half-life	346 h	245–393 h
Main elimination route	Renal	Only 5% is excreted through kidneys
Renal adjustment	Yes (CrCl < 30 mL/min)	No
Hepatic adjustment	No	No
Drug–drug interactions	No	CYP2C9 substrates (warfarin)
Summary of clinical positioning	ABSSSI ± sepsis, but mainly off-label use: deep or complex chronic Gram-positive infections requiring prolonged therapy (osteomyelitis, endocarditis, prosthetic joint infections, and complicated blood stream infection)	ABSSSI, but mainly off-label use: deep or complex chronic Gram-positive infections requiring prolonged therapy (bacteremia, osteomyelitis, endocarditis, and prosthetic joint infections)

**Table 5:** Practical framework for prescriptive appropriateness for the most recent antibiotics in pediatric patients.

Step	Key Question	Prescribing Implication
Confirm infection	Is there clinical evidence of bacterial infection rather than colonization?	Avoid treatment of colonization; obtain cultures and source documentation whenever feasible.
2. Define the pathogen	Which organism and resistance mechanism are present?	Select therapy according to susceptibility and mechanism, not just the organism name.
3. Match drug to site and host	Is the agent suitable for the infection site, the child’s age and weight, the organ function, and the clinical severity?	Optimize dose, infusion strategy, renal adjustment, and monitoring; consider PK/PD support in ICU, ECMO, or CRRT.
4. Justify on-label/off-label use	Is the indication approved? If not, is off-label use evidence-based and documented?	Document rationale, alternatives, expected benefit, risk mitigation, and safety monitoring.
5. Reassess and de-escalate	At 48–72 h, do microbiology and clinical data still support the initial regimen?	Stop, narrow, switch route, adjust dose, and define duration, or escalate only if justified by response and new data.

**Table 6:** Overview of novel antibacterial agents in pediatrics. Pharmacological profile, target pathogens, resistance mechanisms, and pediatric indications of novel broad-spectrum beta-lactams, beta-lactamase inhibitor combinations, and lipoglycopeptides. Abbreviations: ABSSSI = acute bacterial skin and skin structures infection, CAP = community-acquired pneumonia, cIAI = complicated intra-abdominal infection, cUTI = complicated urinary tract infection, EMA = European Medicines Agency, FDA = Food and Drug Administration, GA = gestational age, HAP = hospital-acquired pneumonia, NA = not approved, PNA = postnatal age, RCT = randomized controlled trial, SSTI = skin and soft tissue infection, VAP = ventilator-associated pneumonia.

Drug	Main Target Germs	Main Target Genotypes	PK/PD Index	Approved Pediatric Indications	Age Group	Type of Evidence
Ceftolozane/tazobactam	*P. aeruginosa*, *Enterobacterales*	ESBL	T > MIC	cIAI, cUTI, and pyelonephritis	GA ≥ 32 weeks and PNA ≥ 7 days	RCT
Ceftazidime/avibactam	*Enterobacterales* (*P. aeruginosa* as alternative agent)	AmpC, ESBL, KPC, OXA-48	T > MIC	cIAI, cUTI, and HAP/VAP (EMA only)	>3 months	RCT
Aztreonam/avibactam	*Enterobacterales*, *S. maltophilia* (*P. aeruginosa* as alternative agent)	AmpC, ESBL, KPC, OXA-48, MBL	T > MIC	NA	NA	Ongoing phase II/III trials
Cefiderocol	*Enterobacterales*, *P. aeruginosa*, *A. baumannii,* *S. maltophilia,* *B. cepacia*	AmpC, ESBL, KPC, OXA-48, MBL	T > MIC	NA	NA	Case reports
Meropenem/vaborbactam	*Enterobacterales*	AmpC, ESBL, KPC	T > MIC	NA	NA	Ongoing phase II/III trials
Imipenem/cilastatin/relebactam	*Enterobacterales*, *P. aeruginosa*, *H. influenzae*, *E. faecalis*	AmpC, ESBL, KPC	T > MIC	cUTI and pyelonephritis (in patients with no other option), cIAI (in patients with no other option), HAP/VAP (FDA only)	Birth ≤ 18 years (FDA only)	Ongoing phase II/III trials
Ceftaroline	*Staphylococcus* spp., *Streptococcus* spp., *Haemophilus* spp. (*Enterobacterales* as alternative agent)	MRSA VRE (limited activity)	T > MIC	SSTIs (including MRSA), CAP (not MRSA)	Birth to ≤18 years	RCT
Ceftobiprole	*Staphylococcus* spp., *Streptococcus* spp., *Haemophilus* spp. (*Enterobacterales* and *P. aeruginosa* as alternative agent)	MRSA VRE (limited activity)	T > MIC	CAP (not MRSA)	3 months to ≤18 years	RCT
Dalbavancin	*Staphylococcus* spp., *Streptococcus* spp., *E. faecalis* and *E. faecium*	MRSA, VRE (not VanA)	AUC/MIC	ABSSSI and/or sepsis	Birth to ≤18 years	RCT
Oritavancin	*Staphylococcus* spp., *Streptococcus* spp., *E. faecalis* and *E. faecium*	MRSA, VRE (underexplored)	AUC/MIC	ABSSSI	>3 months	PK/PD study

## Data Availability

No new data were created or analyzed in this study. Data sharing is not applicable to this article.
